# Pre-Infarction Angina and Outcomes in Non-ST-Segment Elevation Myocardial Infarction: Data from the RICO Survey

**DOI:** 10.1371/journal.pone.0048513

**Published:** 2012-12-18

**Authors:** Luc Lorgis, Aurélie Gudjoncik, Carole Richard, Laurent Mock, Philippe Buffet, Philippe Brunel, Luc Janin-Manificat, Jean-Claude Beer, Damien Brunet, Claude Touzery, Luc Rochette, Yves Cottin, Marianne Zeller

**Affiliations:** 1 Department of Cardiology, University Hospital, Dijon, France; 2 Laboratory of Cardiometabolic Physiopathology and Pharmacology, INSERM U866, SFR Santé University of Burgundy, Dijon, France; 3 Department of Cardiology, Clinique de Fontaine-lès-Dijon, Fontaine-lès-Dijon, France; 4 Department of Cardiology, CH Beaune, Beaune, France; S.G.Battista Hospital, Italy

## Abstract

**Background:**

The presence of pre-infarction angina (PIA) has been shown to confer cardioprotection after ST-segment elevation myocardial infarction (STEMI). However, the clinical impact of PIA in non-ST-segment elevation myocardial infarction (NSTEMI) remains to be determined.

**Methods and Results:**

From the obseRvatoire des Infarctus de Côte d'Or (RICO) survey, 1541 consecutive patients admitted in intensive care unit with a first NSTEMI were included. Patients who experienced chest pain <7 days before the episode leading to admission were defined as having PIA and were compared with patients without PIA. Incidence of in-hospital ventricular arrhythmias (VAs), heart failure and 30-day mortality were collected. Among the 1541 patients included in the study, 693 (45%) patients presented PIA. PIA was associated with a lower creatine kinase peak, as a reflection of infarct size (231(109–520) vs. 322(148–844) IU/L, p<0.001) when compared with the group without PIA. Patients with PIA developed fewer VAs, by 3 fold (1.6% vs. 4.0%, p = 0.008) and heart failure (18.0% vs. 22.4%, p = 0.040) during the hospital stay. Overall, there was a decrease in early CV events by 26% in patients with PIA (19.2% vs. 25.9%, p = 0.002). By multivariate analysis, PIA remained independently associated with less VAs.

**Conclusion:**

From this large contemporary prospective study, our work showed that PIA is very frequent in patients admitted for a first NSTEMI, and is associated with a better prognosis, including reduced infarct size and in hospital VAs. Accordingly, protecting the myocardium by ischemic or pharmacological conditioning not only in STEMI, but in all type of MI merits further attention.

## Introduction

Pre-infarction angina (PIA), i.e. angina episodes preceding the onset of acute myocardial infarction (MI), has been suggested in several studies to exert beneficial effects on ST-segment elevation myocardial infarction [1]. In these patients, PIA has been shown to improve the increase in left ventricular wall motion [2], and to induce greater microvascular reflow extent and coronary flow reserve [3]. Moreover, PIA was associated with more rapid reperfusion with thrombolytic therapy [4] and greater degree of ST-segment resolution after primary angioplasty [5]. Several clinical studies reported that PIA both reduces myocardial infarct size [6] and protects against life-threatening ventricular arrhythmias (VAs) [7].

Management of non-ST-segment elevation MI (NSTEMI) patients is a growing clinical challenge, representing nowadays the majority of acute MI in most contemporary registries [8], [9]. Moreover, NSTEMI patients have a dramatically high rate of in-hospital cardiovascular complications, almost similar to STEMI population. NSTEMI are also characterized by increased age, and further evidence of co-morbidities such as diabetes, most conditions that are known to reduce the beneficial effects of PIA in STEMI [10], [11]. However, the impact of PIA in the setting of NSTEMI patients is currently unknown.

From a large contemporary French survey of acute myocardial infarction, the aim of our study was to analyse the frequency and the potential influence of PIA on cardiovascular outcomes in NSTEMI patients.

## Methods

### Patients

The design and methods of RICO (obseRvatoire des Infarctus de Côte-d'Or), a French regional survey for acute MI, have been detailed previously [12]. Briefly, since 1^st^ January 2001, the RICO survey collects data from all the consecutive patients admitted for acute myocardial infarction in all public centres (3) or privately funded hospitals (3) of one eastern region of France (Côte d'Or, 500 000 inhabitants). Between 1^st^ January 2001 and 29^th^ February 2008, all the consecutive patients admitted with a first NSTEMI within 24 hours after the onset of symptoms were included in the present study. MI was diagnosed according to European Society of Cardiology and American College of Cardiology criteria [13]. NSTEMI was defined by the absence of persistent ST-segment elevation or new left bundle branch block on the admission ECG. Patients with documented history of MI were excluded from the study.

### Data Collection

Data were collected at each site by a trained study coordinator using a standardized case report form. Cases were ascertained by prospective collection of consecutive admissions. Eligible patients are identified during the index admission and medical records are reviewed on an ongoing basis after appropriate consent has been obtained. In addition, hospital listings of discharged patients are systematically reviewed to identify eligible cases with use of the International Classification of Diseases (ICD-9), and corresponding codes in ICD 10. Standardized definitions for MI, patient-related variables and clinical outcomes were used. The present study complied with the Declaration of Helsinki and was approved by the ethics committee of University Hospital of Dijon. Each patient gave written consent before participation.

Data on demographics and risk factors (history of hypertension or treated hypertension, diabetes, hypercholesterolemia, current smoking) were collected prospectively, along with admission characteristics and hemodynamic parameters, such as heart rate and systolic and diastolic blood pressure. Height and body weight were self-reported and body mass index (BMI) was calculated (kg/m^2^). Obesity was defined as BMI ≥30. Echocardiography was performed at day 2±1 by a local investigator according to the Simpson method using the apical views to calculate left ventricular ejection fraction (LVEF). Treatments administered before and <48 h after hospitalization were also recorded.

The median duration of stay in intensive care unit was also collected. The Global Registry of Acute Coronary Events (GRACE) score, including admission variables including age, heart rate, serum creatinine, systolic blood pressure, Killip class, cardiac arrest, ST-segment deviation, and cardiac markers, was calculated for each patient (www.outcomes-org/grace/acs_risk.cfm) [14]. Blood samples were drawn at admission. Plasma creatinine levels were measured on a Vitros 950 analyzer (Ortho Clinical Diagnostics, Rochester, NY). Cockcroft-Gault formula was used to estimate serum creatinine clearance. C-reactive protein was measured on Dimension Xpand (Dade Behring, Newark, NE) with an immunonephelometry assay. Plasma N-terminal pro B-type natriuretic peptide (NT-proBNP) was determined by ELISA with an Elecsys NT-proBNP sandwich immunoassay on Elecsys 2010 (Roche Diagnostics, Basel, Switzerland). Plasma troponin Ic and creatine kinase peaks were assessed by sampling every eight hours during the first two days after admission (Dimension Vista Intelligent Lab System, Siemens).

### Coronary angiography

Of the 1541 patients included in the study, 1437(93%) had coronary angiographic data available. Among these patients, most (i.e. 1400/1437 (97%) underwent coronary angiography during their hospital stay and were included in the angiographic analysis. Significant stenosis was defined as a >50% stenosis in an epicardial vessel.

### Outcomes

In-hospital adverse events—i.e. VAs, recurrent MI, cardiogenic shock or death—were recorded. VAs were defined as either sustained ventricular tachycardia (VT) or ventricular fibrillation (VF). VT was defined as a regular wide complex tachycardia of ventricular origin lasting >30 sec or requiring termination due to hemodynamic instability. FV was defined as irregular undulations of varying shape and amplitude on ECG without discrete QRS or T waves that resulted in prompt hemodynamic compromise requiring direct-current cardioversion. Heart failure was defined as a Killip class >1. Recurrent MI was diagnosed by ECG modifications and increased serum troponin.

After hospital discharge, 30-day information on cardiovascular death was acquired by contacting each patient individually, their relatives, or treating physician and by reviewing the hospital records if the patient had been re-hospitalized. Thirty-day follow-up was achieved for most patients (99%).

### Definition of pre-infarction angina

Data were prospectively collected on the study form regarding whether patients have ever experienced angina before acute MI. PIA was defined as patients who experienced typical chest pain, chest discomfort or left arm and jaw pain <7 days before the episode leading to admission, lasting less than 20 minutes and having the same character as the admission episode. Patients were categorized into two groups depending on whether or not they experienced PIA.

### Statistical analysis

Data are presented as median (25th to 75th percentile) or number (percentage). For continuous variables, we used the Kolmogorov-Smirnov analysis to check the normality of the distribution. They were compared using either Student's t test or Mann and Whitney, as appropriate. Dichotomous variables, expressed as numbers and percents, were compared by the χ^2^ test.

Multivariate logistic regression analysis was used to identify independent predictors of PIA on admission. Variables were included in the multivariate model if associated with PIA by univariate analysis (p<0.1), i.e. chronic treatments (aspirin, nitrates and nicorandil), family history of CAD, SBP on admission, obesity and hypertension.

Multivariate logistic regression analysis was used to assess factors potentially associated with the development of in-hospital VAs. The following factors were included: on admission hemodynamic parameters (SBP, heart rate), creatinine clearance, on admission heart failure, female gender, age, and PIA (model 1). In order to analyze the potential role of infarct size on the protective effect of PIA, another model (model 2) was built by adding CK peak to the model 1. Variables entered into the models were chosen based on their significant relationship with VAs in the literature. [15] By using backward selection, only factors with a p value<0.05 were included in the final model. Non-normal variables, such as CK peak, were log-transformed before inclusion in regression analyses. Statistical analyses were performed with SPSS software (SPSS, Inc, Chicago, Ill).

## Results

### Study population

1541 patients were included in the study, of whom 693 (45%) patients suffered from PIA. The patient characteristics are summarized in Table 1. Median age was 69 (56–78) years. Patients with PIA were more likely to have a familial history of coronary artery disease (32% vs. 26%, p = 0.016) and a higher rate of obesity (27 vs. 22%, p = 0.009) than patients without PIA. There was no difference for the two groups for the other risk factors. PIA patients were more often previously treated with K_ATP_ openers (3% vs. 1%, p = 0.005), nitrates (13% vs. 6%, p<0.001) or aspirin (22% vs. 12%, p<0.001). The mean time from symptom onset to admission was similar for both groups (p = 0.26). Moreover, GRACE risk score, heart failure on admission, MI location and LVEF were similar for the two groups.

**Table 1:** Patient characteristics (n (%) or median (interquartile range)).

	No pre-infarction anginaN = 848	Pre-infarction anginaN = 693	p
**Risk factors**			
Age, year	69(55–78)	69(57–78)	0.53
Female	282(33)	211(30)	0.26
Obesity	183(22)	190(27)	0.009
Hypertension	455(54)	406(59)	0.06
Diabetes	196(23)	175(25)	0.36
Hypercholesterolemia	372(44)	316(46)	0.53
Current smoking	226(27)	163(24)	0.18
Familial history of CAD	221(26)	220(32)	0.016
Stroke	48(6)	33(5)	0.50
PAD	77(9)	61(9)	0.92
**Chronic treatments**			
Nicorandil	8(1)	21(3)	0.005
Amiodarone	24(3)	14(2)	0.39
Aspirin	99(12)	153(22)	<0.001
Beta blocker	188(22)	175(25)	0.17
ACE inhibitor	136(16)	113(16)	0.94
Statin	157(19)	138(20)	0.53
Trimetazidine	32(4)	36(5)	0.22
Nitrates	52(6)	93(13)	<0.001
**Clinical data on admission**			
KILLIP >1	155(18)	112(16)	0.31
LVEF, %	60(50–66)	60 (50–66)	0.93
SBP, mm Hg	141(122–160)	144(129–165)	0.09
DBP, mm Hg	80(70–91)	80(70–93)	0.20
HR, b/min	77(65–90)	78(67–90)	0.49
Anterior wall location	303(36)	235(34)	0.49
Time to admission (min)	195(113–420)	210(110–498)	0.26
GRACE risk score	126(98–155)	129(98–156)	0.94
**Biological data**			
CRP, mg/L	6.0(2.3–18)	5.4(2.2–14)	0.14
Creatinine clearance, mL/min	72.3(50.9–94.7)	72.6(50–97.6)	0.44
NT-proBNP, pg/mL	736(229–2172)	813(266–2581)	0.26
Glucose, mmol/L	6.55(5.64–8.48)	6.45(5.54–8.37)	0.14
Troponin Ic, peak >100 ULN	328(39)	211(31)	<0.001
CK peak, IU/L	322(148–844)	231(109–520)	<0.001
**Angiographic data**	**N = 744**	**N = 656**	
Nb diseased vessel			0.001
0	136(18)	73(11)	
1	280(38)	245(37)	
2	182(24)	184(28)	
3 or LM	146(20)	154(23)	
>50% stenosis			
LAD	385(52)	408(62)	<0.001
Cx	346(46)	313(48)	0.651
RC	330(44)	330(50)	0.026
LM	30(4)	49(7)	0.005
**Acute treatments**			
Vasoactive drug	36(4)	20(3)	0.20
Amiodarone	56(7)	33(5)	0.15
Aspirin	771(91)	649(94)	0.059
Beta blocker	659(78)	555(80)	0.29
ACE inhibitor	417(49)	407(59)	<0.001
Statin	545(64)	545(79)	<0.001
PCI	410(48)	402(58)	<0.001

*ACE: angiotensin converting enzyme; CABG: coronary artery bypass grafting; CAD: coronary artery disease;CK: creatine kinase; CRP: C-reactive protein; DBP: diastolic blood pressure; HR: heart rate; LVEF: left ventricular ejection fraction; PAD: peripheral artery disease; PCI: percutaneous coronary intervention; SBP: systolic blood pressure. LM: Left main; Cx: Circumflex; RC: Right coronary; LAD: Left anterior descending.*

**Figure 1:**
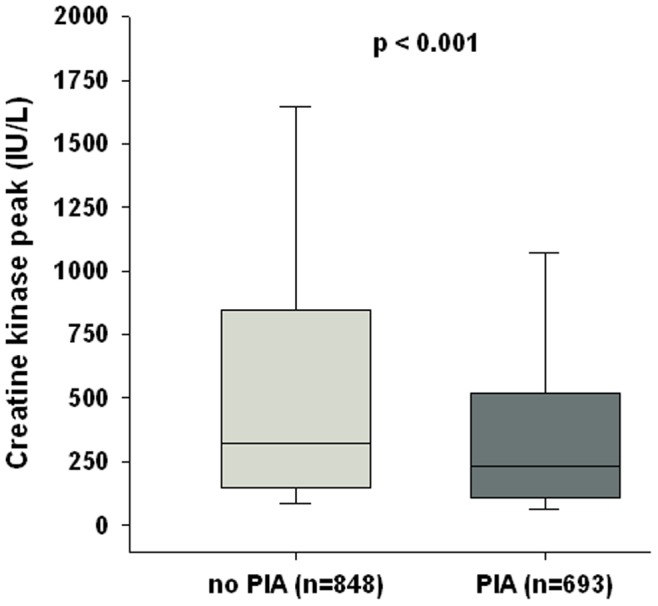
Levels of Creatine Kinase peak (IU/L) during the hospital phase.

PIA was strikingly associated with a lower level of both CK peak (Figure 1), (231(109–520) vs. 322(148–844) IU/L, p<0.001) and troponin Ic peak >100 ULN (211(31%) vs. 328(39%), p<0.001), as a reflection of infarct size. Other biological data, such as CRP, creatinine clearance, NT-proBNP and glycemia on admission were similar for the 2 groups. Within 48 hours after the admission, patients with PIA were more aggressively treated, by either percutaneous coronary intervention (PCI) or acute medications such as ACE inhibitors or statins. On coronary angiography, patients from the PIA group were characterized by less lack of significant stenosis and more frequent significant stenosis on left anterior descending artery or left main (table 1).

By logistic regression analysis (Table 2), preadmission treatment, such as aspirin, nitrates or nicorandil, and family history of CAD were independently associated with PIA.

**Table 2:** Multivariate analysis for predictors of pre-infarction angina.

	OR (95% CI)	p
Aspirin	1.89(1.40–2.55)	<0.001
Nitrates	1.99(1.36–2.91)	<0.001
Nicorandil	2.46(1.01–5.95)	0.046
Family history of CAD	1.27(1.00–1.61)	0.049

*CAD: coronary artery disease.*

### Outcomes

**Figure 2:**
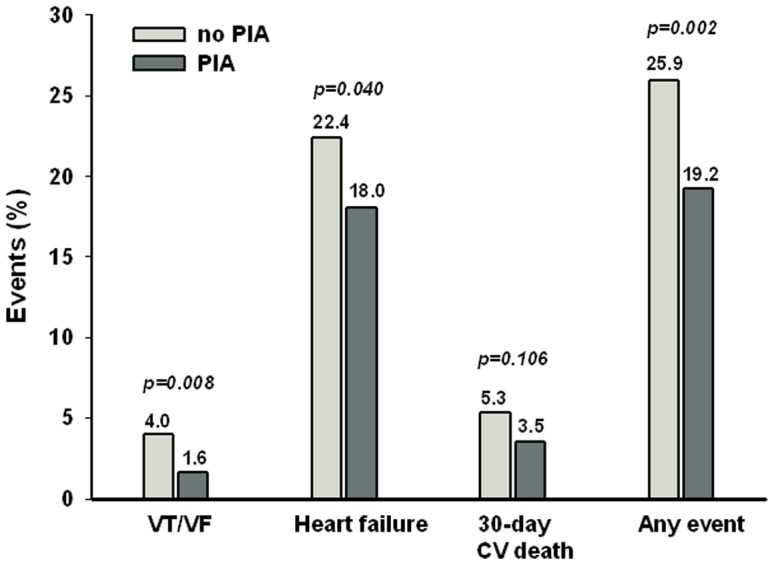
Cardiovascular events (%). *CV: cardiovascular; VT/VF: ventricular tachycardia/ventricular fibrillation*.

Patients who suffered from PIA were markedly less likely, by three fold, to experience VAs during the hospital stay (1.6 vs. 4.0%, p = 0.008) than those without PIA (Figure 2). Moreover, patients with PIA suffered less frequently from heart failure (18.0 vs. 22.4%, p = 0.040). In patients with PIA, there was also a trend toward a decrease in case-fatality at 30 days (3.5 vs. 5.3%, p = 0.106). Overall, a 26% decrease in such short term CV events was reported (19.2% vs. 25.9%, p = 0.002) (Figure 2). The rate of recurrent MI was similar for the 2 groups (p = 0.58).

By multivariate analysis, the presence of PIA was a significant predictor of in-hospital VAs (odds ratio (OR) 0.45; (95% confidence interval (CI): 0.22–0.93; p = 0.03) (model 1, Table 3).

**Table 3:** Multivariate analysis for predictors of in-hospital ventricular arrhythmias.

	Model 1		Model 2	
	OR (95% CI)	p	OR (95% CI)	p
PIA	0.45 (0.22–0.93)	0.030	0.54 (0.26–1.11)	0.100
SBP	0.98 (0.97–1.00)	0.011	0.99 (0.98–1.00)	0.026
HR	1.01 (0.99–1.03)	0.068	1.01 (1.00–1.03)	0.048
CK peak	-	-	2.03 (1.09–3.80)	0.027

*CK: creatine kinase; HR: heart rate; PIA: pre-infarction angina; SBP: systolic blood pressure.*

Subgroup analysis showed that beneficial effects of PIA on VAs tended to be observed in most subgroups (i.e. ≥65 (OR 0.47; 95% CI: 0.20–1.06; p = 0.068) and <65 y (OR 0.29; 95% CI: 0.08–1.03; p = 0.056), female (OR 0.24; 95% CI: 0.05–1.07; p = 0.062) and male (OR 0.47; 95% CI: 0.21–1.03; p = 0.060), with dyslipidemia (OR 0.35; 95% CI: 0.11–1.10; p = 0.072) and without (OR 0.43; 95% CI: 0.18–1.03, p = 0.059) and with or without acute treatment such as PCI, ACE inhibitors or statins. Interestingly, the influence of PIA on VAs was lessened in patients with CV risk factors such as hypertension ((OR 0.91; 95% CI: 0.37–2.23; p = 0.845) vs. (OR 0.12; 95% CI: 0.03–0.51; p = 0.004) without hypertension) or obesity ((OR 0.79; 95% CI: 0.17–3.58; p = 0.758) vs. (OR 0.36; 95% CI: 0.16–0.80; p = 0.012) without obesity), or under chronic use of aspirin ((OR 0.97; 95% CI: 0.16–5.91; p = 0.974) vs. (OR 0.35; 95% CI: 0.16–0.76; p = 0.008 without aspirin) or nitrates ((OR 0.96; 95% CI: 0.13–6.85; p = 0.968) vs. (OR 0.38; 95% CI: 0.17–0.85; p = 0.018) without nitrates). These data suggest that PIA has no additional beneficial effect in patients who have already been protected by such treatment.

Strikingly, when CK peak, as a reflection of infarct size, was added to the model 1, PIA lost its significant association with VAs (OR 0.54; 95% CI: 0.26–1.11; p = 0.10) (model 2, Table 3), suggesting that PIA may limit the development of VAs at least in part through beneficial effects on infarct size. A similar loss of association was found when troponin peak -instead of CK peak- was introduced in the model.

Patients with PIA were more aggressively treated (PCI, CABG, aspirin, statin or ACE inhibitors) that could potentially reduce the incidence of VAs (Table 1). However, logistic regression analysis failed to show any association between these treatments and the incidence of VAs, further suggesting that the beneficial effect of PIA was independent of such treatments (p = 0.955, p = 0.914, p = 0.757, p = 0.967 and p = 0.871 respectively).

## Discussion

To the best of our knowledge, this is the first large prospective study to report that pre-infarction angina in patients admitted for a first NSTEMI 1) is very common, occurring in almost 1 in 2 patients, 2) exerts a beneficial effect on short-term outcomes, especially on VAs and is associated with a smaller infarct size. 3) This beneficial effect is less pronounced in patients with CV risk factors such as hypertension or obesity, or under chronic use of CV drugs such as aspirin or nitrates.

Only small sample size study had analysed the impact of PIA in NSTEMI, suggesting decreased in hospital complications. [16] The high rate of PIA observed in our study (>40%) is consistent with the rate reported in STEMI. [6], [17] In a recent meta-analysis, PIA was observed in 35% of patients presenting a STEMI. [18] A higher rate of PIA in NSTEMI (vs. STEMI) has also been found in previous studies reporting that patients experiencing PIA were more likely to experience NSTEMI than STEMI. [19], [20]. In clinical situations, there is a wide heterogeneity of the timing onset of PIA before the acute MI, ranging from the first 24 hours to 2 months [1], [2]. In agreement with previous works [5], [21], our data strongly support a beneficial and protective role of PIA, when experienced within 7 days before the index event. Patients with PIA had similar risk profile than patients without PIA. However, they were more frequently obese, hypertensive and with family history of CAD, consistent with coronary angiographic findings showing a trend for more CAD extent in such group.

Angina occurring before a first STEMI has been suggested to confer multiple cardioprotective effects. TIMI-4 trial showed a significant decrease in hospital death (3 vs. 8%), severe congestive heart failure or shock (1 vs. 7%), and CK peak determining infarct size (119 vs. 154 IU/L) associated with PIA. [22], [23] TIMI-9 trial further reported that patients with angina onset within 24 hours of infarction had a lower 30-day cardiac event rate (including death, recurrent MI, heart failure, or shock) than those with onset of angina >24 hours (4% vs 17%) [1]. In-hospital VAs are rather uncommon but major life-threatening complication in acute MI [24], [25], [26], in particular in NSTEMI [15]. However, only few trials have assessed the impact of PIA on such arrhythmias, limited to out-of-hospital arrhythmias [7] or reperfusion arrhythmias [25]. In our work, PIA was associated with a decreased infarct size -by 28%, as measured by CK peak-, consistent with previous findings [1], [4], [23]. Our work also showed that conditioning the heart can confer additional benefit over current medical practice procedures. Moreover, our results from multivariate models showing a loss of prognostic capacity of PIA when CK was added to the model, interestingly suggest that PIA may have contributed to the decreased incidence of VAs, via a lower infarct size. However, the underlying mechanisms of the beneficial effects of PIA are not yet clarified.

The PIA-induced development of coronary collateral circulation from the non-ischemic areas has been suggested. Some authors also proposed that increases in pressure due to a subtotal occlusion during short episodes of angina could play an important role by opening and developing coronary collateral vessels, especially in diabetic patients [27]. However, in contrast to experimental studies, the involvement of coronary collateral circulation in the cardioprotective effect of PIA in humans remains controversial. In patients undergoing PCI, an antiarrhythmic effect of preconditioning can occur independently of collateral recruitment [28] Moreover, the protective role of PIA has been observed even in the absence of significant collateral circulation [2], [29]. In NSTEMI patients, where coronary arteries are not totally occluded, the involvement of such pathophysiological mechanism in the beneficial effects of PIA may be only modest.

Another potential cardioprotective mechanism relates to experimental ischemic preconditioning. Preconditioning the myocardium during brief episodes of ischemia, before a sustained occlusion, stimulates adenosine receptors, decreases the cellular influx of calcium, leading to a decrease in myocardial energy demands and limiting the extent of myocardial injury. [30] Transient mitochondrial permeability transition pore (mPTP) opening mediates preconditioning-induced protection, via a K+ ATP-dependent channel [31]–[33]. Experimental preconditioning has been shown to typically reduce infarct size and decrease in ischemia-reperfusion arrhythmias in most animal models. [34]–[36] Ischemic preconditioning could also induce antiarrhythmic protection in humans [37]–[41].

Finally, chronic treatment with CV drugs such as aspirin or nitrates, taken before the acute MI, may improve outcomes in patients experiencing PIA. The cardioprotective effect of such chronic preadmission treatments has been indirectly suggested, in a recent retrospective work showing that patients under chronic CV treatments (i.e. aspirin, β-blockers, ACE inhibitors, or statins) before hospital admission were less likely to develop STEMI than NSTEMI. [19] Interestingly, the risk proportionally decreased with the increasing number of medications used before acute MI, underlining the benefit of preventive medication in high-risk patients. Moreover, in the GRACE Study, a history of angina was more common among patients with NSTEMI than among those with STEMI, further lending support for the hypothesis that prior treatment may also modify the disease process and clinical presentation [20]. Nitroglycerin conferred cardioprotection against ischemia through a protein kinase C-dependent pathway [42], [43]. Recently, in a large multinational, unselected population over 50.000 MI patients, chronic nitrate use pre-infarction was associated with significantly lower levels of cardiac markers of necrosis, further suggesting a smaller extent of myocardial necrosis [44]. Hence, in our study, such treatments may have participated at least in part to the beneficial effect associated with PIA.

Our findings on the attenuation of the cardioprotective effect associated with PIA in some subgroups, such as obese or hypertensive patients is consistent with previous works [45], [46]. The persistence of myocardial preconditioning in older patients is controversial [10], [47]. Our data interestingly suggest that beneficial effects of PIA could be maintained in the older (>65 y) NSTEMI patients.

## Conclusion

Our study providing for the first time evidence a beneficial effect associated with PIA in patients presenting a NSTEMI, extent the findings from small proofs-of-concept studies in STEMI patients to all types of MI, on the potential clinical benefit of conditioning the myocardium. Recent randomized trials showed that promising therapeutic intervention i.e. remote ischemic conditioning could exert cardioprotective effect independently of occluded vessels, and suggest mechanisms underlying such benefit at the cellular levels, beyond restoration of perfusion [48]. Accordingly, protecting the myocardium by ischemic or pharmacological conditioning not only in STEMI, but in all type of MI merits further attention.
